# Analyzing the social context of health information and misinformation during the COVID-19 pandemic: a case of emerging inequities in Lebanon

**DOI:** 10.1177/1757975920984178

**Published:** 2021-03

**Authors:** Jihad Makhoul, Tamar Kabakian-Khasholian, Lea Chaiban

**Affiliations:** Department of Health Promotion and Community Health, Faculty of Health Sciences, American University of Beirut, Beirut, Lebanon

**Keywords:** communication, social marketing, education campaign, media communications, Middle East, communities, health promotion

## Abstract

With the far-reaching COVID-19 pandemic starting in December 2019, a surge of misinformation, now coined by the World Health Organization (WHO) as an ‘infodemic’, has also taken the world by storm. False information and variations in interpretations about the pandemic and mitigation interventions/strategies continue to spread at a faster pace than the relevant scientific evidence. The WHO has called for a fight against this infodemic, describing it as the most contagious aspect of the pandemic. In this era of rapid information exchange, public health measures, and state interventions to control the pandemic, a contextual understanding of how information is communicated and shared is important for uncovering possible reasons for action or inaction by the general public. With the Lebanese state scrambling to implement and enforce different measures to control and mitigate the spread of COVID-19, adherence by the general public is not uniform. In this paper, we refer to social science and risk communication theory to discuss how the political, economic and social contexts in the country, and not only the content of the messages that people receive from officials, affect how they interpret and act on information. We highlight how this has played out in Lebanon and identify societal aspects of importance in a low-middle income country fraught with social, economic and political inequalities which continue to undermine the efforts to contain the spread. Implications to inform state response in the context of low-middle income countries are also discussed.

## Introduction

The spread of misinformation related to a public health issue is not new, and has become more prominent and rapid with the global proliferation of social media. The ongoing COVID-19 pandemic that started in December 2019 has been accompanied by an unprecedented surge of misinformation that has been described as being as harmful as the pandemic itself. Misinformation is false information that is spread regardless of its intent to mislead, and its spread causes an infodemic. The World Health Organization (WHO) has called attention to the infodemic accompanying the COVID-19 pandemic, explaining that an excessive amount of information circulating globally is spreading misinformation and rumors, and is impeding public health responses (1). The WHO Director-General pointed to the important and debilitating role of this infodemic, and called for a fight against it by stating that ‘misinformation on the coronavirus might be the most contagious thing about it (2)’. The rapidly circulating misinformation on the pandemic can be twisted or fabricated intentionally or unintentionally. An analysis of a sample of social media posts in English, for example, has found that much of the pandemic-related information posted on a sample of social media, and which had the highest engagement, was reconfigured or reworded (3). The same study found that false statements were made by politicians, high-level elected officials, celebrities, and other prominent public figures speaking publicly or to the media, and by the general public about disease spread and remedies, such as saunas, hair dryers and exposure to the sun preventing COVID-19; or images and videos described differently than what they were intended to convey (3).

The non-pharmaceutical interventions of physical distancing, self-isolation and self-protection guidelines are the main public health measures that are being adopted in the global fight against the COVID-19 pandemic. These are all dependent on behaviors to reduce the spread of the virus. Effective health communication strategies are therefore necessary, as is the utilization of risk communication for many reasons: to provide useful and applicable health-related information, to address circulating rumors, and to diffuse a narrative that supports the public health measures put in place.

Risk communication in public health is based on behavioral and social science theory, practice and research, and aims at promoting healthy behaviors among individuals and communities through strategized exchange of information. It is considered an integral part of public health preparedness plans and emergency responses (4). Two models inform risk communication: the realist approach, which considers risk to be objective and independent of its social context, and the social constructionist approach, which assumes risk is constructed through social and cultural processes. This second approach is recognized to better reflect individual and community views of risk, and is useful to explain how communities act, adopt mechanisms to reduce risk perception, and control anxieties (5,6).

On 15 March 2020, the Lebanese government declared a ‘state of general mobilization’ after reporting its first confirmed COVID-19 case on 21 February 2020. Since then, the country has been experiencing an increase in positive cases which pushed the authorities to extend the lockdown more than five times (7). Citizens have been obliged to abide by strict public health measures of nighttime curfews and vehicle movement rotation based on odd–even plate numbers (8). The Lebanese state has been struggling since then to reach and maintain public adherence to these measures, especially in view of the gradual ease of the lockdown that started in May 2020. The drop in numbers of COVID-19 cases encouraged the government to progressively loosen up lockdown measures, and partially reopen public spaces, which has led to a spike in numbers, registering 71 new cases on 10 July 2020, its highest. As of 10 July 2020, Lebanon reported 2012 cases, 1368 recovered, and 36 deaths (9).

Within these efforts and as part of its public health response, the government’s message-centered approach to health communication relies largely on a uni-directional and didactic provision of information that addresses self-protection measures and the importance of flattening the curve. This realist approach in risk communication allows for misinterpretation of risk in the midst of the scientific uncertainties about the disease and the new information related to the mode of transmission, and recommendations about protection measures. In local and global situations of rapid information exchange, a contextual understanding of the public’s perception of risk and interpretation of messages is necessary to guide public health actions and interventions during this pandemic. Research on the social determinants of health has established that people’s behaviors and health outcomes are influenced by a myriad of conditions at the interpersonal, community and societal levels. These conditions can act to either support health-protective behaviors, such as providing enabling and supportive environments for people to make healthy choices; or can also create obstacles or exacerbate existing inequalities to adversely affect health outcomes (10).

Several factors in the Lebanese context conspire to affect people’s actions or inactions in the face of the COVID-19 pandemic. These same factors play a decisive role in the course of this pandemic in similar multicultural settings around the globe (11). Problematic or inadequate use of evidence, contradictory official messages, and a vast spread of misinformation, on the one hand, as well as contextual factors within which these messages circulate, influence how people access, interpret and act on information. In this paper, we argue that the population’s access to, interpretation of, and action on information are affected by the collectivist nature of society, the dire socioeconomic conditions of the country, a history of state failures, and a stark general mistrust in the national health system. By using the example of Lebanon, and referring to the review of evidence by Van Bavel *et al*. (11) which follows a social and behavioral science lens to analyze a selection of research topics relevant to pandemics, we demonstrate the need for integrating contextual considerations in designing messages and engaging with various communities. This is necessary to allow the public an opportunity to accept and implement public health measures for the management and control of outbreaks. The analysis of the experience in Lebanon is relevant to public health responses in similar contexts around the globe. With increased global connectedness, societies are in constant change and portray various forms of internal diversity; thus lessons learned from such experiences can inform stakeholders in designing future health communication for similar responses.

## Societal values and social ties

Social network analysis (SNA) of infectious diseases, and in particular of respiratory diseases, has found that these infections are likely to spread in social networks where social norms among group members are shared (12). SNA has been shown to be successfully applied in sexually transmitted infections, tuberculosis (13,14), and in Severe Acute Respiratory Syndrome (15). In these social networks, individuals’ perceptions of what behaviors are accepted or not by a particular group will influence the adoption of these behaviors, which can be either health protective or health damaging (16). A network analysis of Middle East Respiratory Syndrome has identified the patients who were the most influential in spreading the infection, and used the location of these patients in predicting individuals who will most likely be infected (17). Social networks are used to disseminate health information through trusted sources, such as a local radio station in Nepal that improved contraception adoption within social networks (18), and community networks have had a role in disseminating HIV-related messages and improving the adoption of preventive measures (19,20).

In collectivist cultures, such as that of Lebanon, social relationships are dense and manifest in different ways in the lives of people through tribal, immediate family, and extended family networks and beyond. Lebanese society is predominantly a strong kinship culture, which resonates with many multicultural settings around the globe and is in contrast to individualist cultures (21). People have relied on family structures for economic support and security ever since the formation of the Lebanese state in the 1920s (22). Extended family structures and fictive kinship continue to permeate society, and influence individual behaviors and desires (21,23). This social setting has influenced the spread of both harmful and beneficial behaviors during the pandemic. Localized spread of the virus was reported from several villages in Lebanon where social ties between residents are strong. The communal living arrangements of extended families, who often live in close proximity to each other, have posed an obstacle for the practice of social distancing. Communal celebration of good news, such as visiting community members returning from travel, and information spread about PCR test results for COVID-19 are common practices. In one instance, a community member from a village in North Lebanon whose PCR results turned out positive after an earlier false negative was a potential source of infection to those who visited him at home (24). Both harmful and beneficial behaviors were also noted in the mountain town of Bsherri, a remote Lebanese rural community, which was identified with a cluster of COVID-19 infections in April 2020. The spread of the infection was attributed to social visits among extended family members and the difficulty of observing precautionary measures, such as physical distancing, in this collectivist community (25). These same close community ties have been instrumental in mitigating the spread of infection. The community mobilized itself and abided by the instructions given by the local municipality and the ministry officials, and was able to limit the spread by following the strict measures of quarantine, travel restrictions and night curfews. The local public hospital set up a response team, and accommodated positive cases (26,27), while the villagers who were not residing there at the time mobilized to provide moral support to the community through social media (28).

Similarly, the notion of personal space in collectivist societies differs from that in individualist societies. Triandis (29) explains that privacy and personal space are often much valued among individualist societies where people should think freely and mind their own business. Consequently, invading other persons’ space is disrespectful and rude. Collectivists are more open, are less conscious of other people’s spaces, and expect others to unveil more about themselves as members of a group. Unlike individualists who value independence and autonomy, and whose social behaviors are based primarily on personal attitudes, collectivists’ behaviors are shaped by group norms and are interdependent within their in-groups (30,31). In collectivist societies, personal space is minimal, and those who refrain from interacting with people in a collective space are frowned upon. This has a bearing on the physical distancing recommendation, which is difficult to understand and to apply in Lebanon because this concept is alien to this culture.

## Political influences

In Lebanon, trust in the public sector and the state is frail and continues to erode. Historically, and up until the establishment of the Lebanese state in 1920, Lebanese people have held allegiance to their many religious sects, which have been allowed by the constitution to be represented in the state through allocating state leadership positions, such as the president, speaker of parliament and prime minister, to religious sects (32,33). When a leader gains power in social, political and economic spheres, other members expect to receive a distribution of these resources. Even though the state allows elections for political representation, elections also allow sectarian representatives a chance to continue their power, because their family members and loyal supporters elect them into position, creating political rivalry between respective families (34). The collapse of the Lebanese state in various periods of modern history, such as during the Lebanese Civil War (1975–90), has created a vacuum which warlords filled. They have ruled the country since 1990, creating instead of a trustworthy egalitarian system, an unequal system reinforcing clientelism and social inequity which reproduced the same political-sectarian system that led to the recent collapse of the national economy (35). During the lockdown, and amidst the social and economic problems in the country and dwindling resources to respond to the pandemic, political parties provided services to the public. These included disinfecting hotels allocated for quarantine, and distributing food rations and personal items to the Lebanese returnees lodging there, which could be an attempt to take advantage of vulnerabilities for political gain. This disaggregate approach to handling the outbreak in the country has further diminished the role of the state and contributed to heightening public feelings of distrust in the government. For example, the government’s use of ‘general mobilization’ measures in the country has been described by protestors as an attempt to crack down on freedoms and to tighten its grip on the streets which witnessed protests, while security forces forcibly removed and arrested the remaining few protestors (36). The lack of adherence to messages of self-protection disseminated by the official governmental channels may have been made worse by people’s disbelief that the pandemic is real. This was reinforced by local media reports of crowding on public beaches, and people’s refusal to take voluntary PCR tests because of their belief in a conspiracy in which the situation was thought to be created by the state to attract relief funding for the country’s economic problems. In times of pandemics, trust is an important cue for selecting the persons or organizations that lay persons rely on to make decisions. Trust is consequently an important factor in risk management (37). Delayed risk communication and contradictory messages and their enactment by the Lebanese government officials affected people’s trust. Daily television appearances as they are surrounded by their entourage on official business; handling facial masks when speaking to reporters in close proximity; and celebratory gatherings to honor politicians, are all examples of contradictions to the verbal messages of physical distancing and use of facemasks by the general public.

## Social inequalities and multiple vulnerabilities

In Lebanon, disadvantage and marginalization are not new, and are widespread among both Lebanese and non-Lebanese people in the country. Lebanon’s already catastrophic economic situation has been made worse by nationwide coronavirus lockdowns. The Lebanese pound has been in freefall for months. The extreme and absolute poverty line estimations for Lebanon have changed dramatically since the 1990s. A family of five was estimated to need approximately US$306 per month (absolute poverty line) to meet its food requirements, while US$618 was estimated to be the extreme poverty line, below which an average family could not meet its education and healthcare needs. An estimated 28% of the Lebanese families (one million people at that time) was described to live below the absolute poverty line, and 7% (250,000) in extreme poverty (38). At that time, 75% of the families who lived from agriculture were poor and 40% extremely poor (38). According to World Bank estimations in April 2020, Lebanon’s current economic crisis could put 22% of the Lebanese population (850,000 individuals) under the extreme poverty line, and 45% (1.7 million individuals) under the upper poverty line (39). Many of these are daily and agriculture workers. In addition to impoverished Lebanese, many other groups suffer from varying degrees and types of disadvantage in the country. Syrian, Palestinian and Iraqi refugees, migrant workers, and incarcerated individuals experience marginalization and multiple social inequalities amidst the absence of equitable social protection schemes. Lebanon hosts 1.5 million Syrian refugees, 13,500 Iraqis, and more than 200,000 Palestinian refugees under UNRWA’s mandate (40), all living in dire socioeconomic conditions. The Lebanese state issued new residency regulations that have made it difficult for Syrian refugees to renew their legal documents and consequently find work. This has forced them to rely on child labor as a sole source of income (41). Similarly, for over 60 years, Palestinian refugees in Lebanon have been denied basic social and civil rights, and have been excluded from participating in the Lebanese labor force and restricted from employment in medical and health professions (42). Welfare, education and healthcare services are offered mainly by the UNRWA and Palestinian agencies in the camp, depending on the availability of funds. Along the same lines, migrant domestic workers in Lebanon experience social exclusion and inequitable access to healthcare services (43).

Like many Arab countries, Lebanon is not a signatory to the 1951 Geneva Convention on the protection of refugees; consequently, Lebanese law treats refugees and asylum seekers as illegal foreigners, and they are consequently subject to arrest, detention, and deportation (44,45). The absence of nationwide plans to ease the economic burden before and during the lockdown, as well as the absence of national preparedness for such emergencies, have exacerbated existing inequalities. For these resource-poor populations, the lockdown means inability to provide food for themselves and their dependents if they are daily workers. Staying at home is not an option for them. The pressing contextual conditions prevent these populations from exercising the necessary directives of social distancing and personal hygiene put forth by state officials. A number of municipal councils have introduced restrictions on Syrian refugees as part of their measures against COVID-19 despite no confirmed cases among refugees at the time. Restrictions to their mobility enforced by the local police exceeded the curfew hours announced by the government, and there was permission to perform only ‘necessary’ tasks, with the threat of confiscation of their identity documentation. These discriminatory measures elevated the refugees’ concerns about their ability to access health care and information on how to protect themselves against the infection amidst the delayed coordination between the government and the refugee service providers, the UN agencies in Lebanon (46).

In addition, Lebanon houses more than 250,000 migrant workers who come from African and Asian countries to work in households, local business and industries (47). These workers are excluded from the Lebanese Labor Law, and are governed by the sponsorship of the ‘*Kafala*’ system in Lebanon, which allows the guarantors to decide on migrant workers’ conditions, putting them at risk of exploitation and abuse (48). There were recent reports on 16 cases of Bengali migrant workers in a detention center owned by a waste management plant, in addition to a similar building run by the Philippine Embassy for Filipino workers who were abandoned by their guarantors. The Bangladesh Embassy Lebanon Facebook page announced that the total number of Bengali nationals who tested positive was 45, and uploaded information in Bengali explaining how COVID-19 is spread, its symptoms, precautionary instructions and the ministry hotline number (49). The embassy posts updates on all Lebanese cabinet’s announcements concerning COVID-19, and has also raised the issue of misinformation, advising Bengalis not to share ‘strange’ voice messages. On 8 and 19 March 2020, the Philippine Embassy posted on its Facebook page simple self-protection instructions in their native language, to protect Filipinos in Lebanon from COVID-19 (50). Although a much-needed initiative, its reach within these groups cannot be determined, given the possibility of restricted access to the internet that they might be subjected to by their guarantors. It is not uncommon to find that prisons holding Lebanese, refugees and migrant workers are congested and have unhygienic conditions, as reported by human rights groups and the Lebanese local syndicate of lawyers. The largest detention site has more than 4000 detainees settled in crowded, unventilated unsanitary cells (51). Amnesty International and the Committee of the Families of Prisoners in Lebanon reported that 120 inmates use the same bathroom, and corridors are sleeping rooms for 70 prisoners at a time. The same prison has 700 prisoners with health conditions, many of whom suffer from respiratory illnesses (52). UNODC Regional Office for the MENA region conducted awareness-raising campaigns in two of the prisons for women and girls (UNODC) (53). However, despite this information, given the dire conditions they are in, it is impossible for prisoners to practice physical distancing.

## The way forward

Throughout this paper we have illustrated how a realist approach to communicating information about emergencies without considerable attention to the context within which this information travels and is used minimizes the effectiveness of risk communication. These contexts differ with variations in social, economic and political conditions which, when not considered in the development of the content and dissemination of information, hamper people’s implementation of recommended strategies. Similarly, the importance of considering the environment in which the behavior change is expected to happen is at the core of many behavior change theories, explaining how information alone is not sufficient for people to change their behaviors (54). The understanding of the audience profile is expected to guide health communication programs and activities, and to inform the contextualization of the messages to enhance public health measures (55,56). The COVID-19 pandemic required an urgent response; however, multiple messages addressing self-protection were developed and disseminated in Lebanon, and elsewhere in the world, with no consideration of the complexities involved in the implementation of the recommended behavior in the message (57,58). What does social distancing mean to a migrant worker detained in a crowded space? How can a refugee abide by recommendations of frequent hand washing? How would refraining from engaging in routine interactions for lengthy periods of time be interpreted by close-knit extended families in rural areas? These questions indicate aspects of context that cannot be addressed with simplified messages requiring specific changes in people’s behavior.

Risk communication highlights the role of community engagement in the design, implementation, and the monitoring and evaluation of communication activities and interventions. It is, therefore, very necessary to identify lessons learned during the COVID-19 pandemic from the perspective of a low-middle income country in order to inform continuing efforts of communicating with the public in the presence of the threat of new waves, a resurgence of the pandemic, or future unforeseen outbreaks. The differences in the public’s experiences with and the use of the communicated messages during this pandemic point to the need to acknowledge variations in the types of societies, and to understand the inherent nature of their social networks and the social relations among their members in order to improve the messages to suit their needs (11). Similarly, existing social structures, social institutions and key individuals, such as heads of extended families, local leaders, or mayors, could be leveraged to mobilize communities for action from within and work out contextually acceptable alternatives to physical distancing and crowding. The state should work on a communication and implementation strategy to provide clear, consistent messages, and to be transparent about what health and other officials know and do not know, with the aim of preventing misinterpretation or misinformation spread. In this regard, the use of the inoculation theory in communication can build resistance to attempts to spread conspiracies and misinformation by exposing the public to such false arguments and giving them the counter-arguments for rebuttal (59). Simultaneously, consistent messages need to be prepared, to be communicated and enacted by state officials to increase trust in state authorities (4,60,61), such as proper use of face masks in public and refusing to attend, or discouraging, celebratory gatherings. The state needs to have a central role in leading on all response preparations, coordinating efforts to avoid creating a vacuum for competing political parties to fill. Most importantly, the state needs to address social inequalities and to provide social and health protection schemes for all its population, particularly for disadvantaged populations, to mitigate economic vulnerabilities. Similarly, the physical and social conditions of jails need improving to enhance personal hygiene. All these interventions will support adherence to or implementation of precautionary measures.
